# Serglycin Cooperates with the Unfolded Protein Response Pathway and Inflammation to Drive Glioblastoma Cell Survival

**DOI:** 10.3390/cells15080660

**Published:** 2026-04-09

**Authors:** Eleftherios N. Athanasopoulos, Chrysostomi Gialeli, Angeliki Natsiou, Dimitra Manou, Vassiliki T. Labropoulou, Achilleas D. Theocharis

**Affiliations:** 1 Biochemistry, Biochemical Analysis and Matrix Pathobiology Research Group, Laboratory of Biochemistry, Department of Chemistry, University of Patras, 26504 Patras, Greece; lefterhsathanasopoulos@gmail.com (E.N.A.); aggelikinatsiou@outlook.com (A.N.);; 2 Cardiovascular Research Translational Studies, Clinical Sciences Malmö, Lund University, 22100 Lund, Sweden; chrysostomi.gialeli@med.lu.se; 3 Hematology Division, Department of Internal Medicine, University Hospital of Patras, University of Patras, 26504 Patras, Greece; vaslabrop@upatras.gr

**Keywords:** glioblastoma, extracellular matrix, serglycin, unfolded protein response, proteoglycans, apoptosis, inflammation

## Abstract

Serglycin (SRGN) has been found overexpressed and secreted in glioblastoma (GBM), associated with tumorigenic signaling and poor prognosis. In this study, we aimed to elucidate the involvement of SRGN in the unfolded protein response (UPR), an oncogenic signaling pathway implicated in protein recycling and cell fate. Herein, we developed stably transduced LN-18^shSCR^ GBM cells, expressing high levels of SRGN, and SRGN-depleted LN-18^shSRGN^ cells. We observed significantly attenuated expression and activity of all UPR mediators upon SRGN suppression, in particular PERK, IRE1, ATF6 and downstream effectors. SRGN-expressing cells possessed a constitutively active UPR, as indicated by its active phosphorylation status and accumulated pool of nuclear ATF4 in LN-18^shSCR^ cells. Constitutive activation of the caspase-dependent apoptotic pathway was apparent in LN-18^shSRGN^ cells. Induction of endoplasmic reticulum (ER) stress pointed out that LN-18^shSRGN^ cells were predisposed to ER stress-associated cell death, whereas LN-18^shSCR^ cells activated adaptive UPR signaling and displayed resistance to apoptosis. The evaluation of TLRs, TNFRs, ILs and NF-kB also underscored that SRGN is essential for their expression and active inflammatory signaling. We concluded that SRGN-expressing cells acquire a pro-survival UPR mechanism, highlighting the novel regulatory role of SRGN in the adaptation and survival of GBM cells.

## 1. Introduction

Glioblastoma multiforme (GBM) describes the most aggressive brain tumor and lethal malignancy of the central nervous system, with a life expectancy of less than 16 months in adults [1,2]. Isocitrate dehydrogenase wildtype (IDH-wt) GBM tumors are categorized in Grade IV diffuse gliomas with high infiltrating properties, that arise from astrocytic neoplasms of the temporal or frontal lobe of the brain. Based on molecular characteristics, GBM is classified as classical, neuronal, and mesenchymal, with epidermal growth factor receptor (*EGFR*) mutations being present in the majority of patients with classical GBM, and *TP53* or platelet-derived growth factor receptor A (*PDGFRA*) mutations mainly observed in cases of neuronal gliomas [2,3]. A combination of chemotherapy, radiotherapy and surgical intervention is rarely sufficient to treat high grade gliomas and is accompanied by crucial side effects and poor prognosis, with high rates of recurrence [4,5].

Among other factors, GBM progresses through multiple alterations of the tumor microenvironment (TME). One of the most prominent non-cellular components of the TME is the extracellular matrix (ECM), a complex macromolecular network upon which cells reside and communicate [6,7,8]. The function of the ECM is not limited to a structural scaffold for tissue organization and integrity, but also expands to plethora of phenomena, including the regulation of cell signaling and homeostasis, adaptation to external pathophysiological stimuli, tumor formation and angiogenesis. To substantiate cell-cell interactions and crosstalk, different cell types secrete diverse biomolecules within the ECM, such as growth factors (GFs), cytokines, proteases, glycoproteins, glycosaminoglycans (GAGs), and proteoglycans (PGs). Proteoglycans consist of a protein core decorated with a variety of GAG combinations, leading to the formation of pleiotropic biomolecules with distinct functions, depending on the type and physiology of the tissue [9,10,11,12]. The tissue that supports neuronal integrity is constructed predominantly by glial cells, hyaluronan (HA), GAGs, and PGs, while tumor-associated brain tissue is further enriched with secreted PGs and glycoproteins [13,14].

Serglycin (SRGN) is the only insofar characterized intracellular PG, while also being secreted within the ECM. It has been found overexpressed in hematopoietic and immune cells, present within secretory granules for regulating their cargo and its bioavailability [15,16]. It has become increasingly evident that malignant cells, such as breast and lung, GBM and multiple myeloma (MM) cells, express and secrete high levels of SRGN, while SRGN has also been proposed as a potential biomarker for GBM, since its’ expression is positively correlated with GBM Grade [17,18,19,20,21,22,23]. Numerous studies have pointed out that SRGN participates in the aggressiveness of tumor cells through inducing oncogenic signaling and regulating both gene expression and ECM remodeling [24,25,26].

Upon malignant transformation, the endoplasmic reticulum (ER) experiences stress, known as ER stress, due to the accumulation of unfolded and misfolded proteins in the ER lumen [27,28]. The demand for excessive cell proliferation and protein turnover results in the activation of the unfolded protein response (UPR) pathway, a protein recycling mechanism that safeguards cell homeostasis, adaptation, and survival [29]. UPR has also been identified as a tumorigenic signaling system in many malignancies, mediated by three ER-resident transmembrane sensors: double-stranded RNA-activated protein kinase (PKR)-like ER kinase (PERK) or EIF2AK3, inositol-requiring enzyme 1 (IRE1) or ERN1 and activating transcription factor 6 (ATF6). The binding immunoglobulin protein (BiP) or HSPA5 chaperone acts as a ‘switch’ by dissociating from the above receptors under ER stress conditions, leading to their activation [30,31,32,33,34,35,36,37]. Each UPR branch initiates a signaling cascade that orchestrates gene expression and events related to protein folding, degradation, inflammatory responses, autophagy, caspase-dependent apoptosis, and eventually cell fate [38,39,40,41,42,43].

Herein, we evaluated the role of SRGN in regulating the UPR mechanism and related signaling networks. We utilized LN-18 and U251-MG GBM cell lines, with the former expressing high levels of SRGN, and proceeded with suppression of SRGN expression. Less aggressive SRGN-suppressed cells exhibited a differentiated phenotype, diminished levels and attenuated activity of UPR markers, accompanied by loss of inflammatory mediators’ activity. Simultaneously, SRGN-depleted cells acquired death-prone properties upon ER stress induction, as indicated by apoptotic markers’ activity and decreased cell viability. By utilizing UPR inhibitors, we also demonstrated that SRGN-expressing cells maintain an active pro-survival UPR machinery to promote cell adaptation and survival under prolonged ER stress.

## 2. Materials and Methods

### 2.1. Cell Culture, Transfection and Reagents

The LN-18 GBM cell line was obtained from the American Type Culture Collection (ATCC). Transduction of LN-18 glioblastoma cells with shRNA lentiviral particles against human serglycin (LN-18^shSRGN^) or scramble shRNA lentiviral particles (LN-18^shSCR^) was performed according to manufacturer’s protocol, as described previously [20]. Puromycin dihydrochloride, at the optimal concentration 0.8 μg/mL (sc-108071, Santa Cruz Biotechnology, Inc., Heidelberg, Germany) was used for the selection and manufacture of stably transduced LN-18 cells. Puromycin-resistant cells were cultured in a humidified 95% air/5% CO_2_ incubator at 37 °C in Dulbecco’s Modified Eagle’s Medium (DMEM) supplemented with 5% (*v*/*v*) heat-inactivated Fetal Bovine Serum (FBS), 1 mM sodium pyruvate, 2 mM L-glutamine and a cocktail of antimicrobial agents (100 IU/mL penicillin, 100 μg/mL streptomycin, 10 μg/mL gentamycin sulfate and 2.5 μg/mL amphotericin B). Human GBM U251-MG cells (kindly provided by Paraskevi Heldin, Uppsala University, Sweden) were cultured with RPMI-1640 GlutaMAX-I, supplemented with 10% (*v*/*v*) heat-inactivated FBS, 100 IU/mL penicillin and 100 μg/mL streptomycin cocktail. All the above reagents, as well as the cell detachment solution [0.05% (*w*/*v*) trypsin in PBS/0.02% Na_2_EDTA] were purchased from Biosera, Chole, France. Tunicamycin (TM), an N-glycosylation inhibitor (Sigma-Aldrich, St. Louis, MO, USA), was used as an ER stress inducer and was resuspended in DMSO. UPR Inhibitors MKC8866 IRE1 RNase inhibitor (S8875), Kira 6 IRE1 kinase inhibitor (S8658), GSK2606414 PERK kinase inhibitor (S7307) and Ceapin-A7 ATF6 inhibitor (E1099) were purchased from Selleck Chemicals, Houston, TX, USA and resuspended in DMSO. ON-TARGETplus SRGN siRNA 5 nmol (L-019692-00-0005) for transient SRGN silencing, ON-TARGETplus Non-targeting Pool 5 nmol (D-001810-10-05) negative control, DharmaFECT 1 Transfection Reagent (T-2001-02), 5X siRNA Buffer (B-002000-UB-100), and Molecular Grade RNase-free water (B-003000-WB-100) reagents were purchased from Dharmacon Inc., Lafayette, CO, USA, HVD Biotech Vertriebs Ges.m.b.H, Vienna, Austria. siRNAs were resuspended with 1X siRNA Buffer and treatment solution included Opti-MEM (Gibco, #31985062, Thermo Fisher Scientific Inc., Waltham, MA, USA). The working siRNA concentration was 50 nM and the protocol was performed according to manufacturer’s protocol.

### 2.2. RNA Isolation, cDNA Synthesis and Real-Time qPCR Analysis

LN-18 cell lines were seeded in 60 mm dishes and grown in complete medium up to 70–80% confluency. Cells were starved overnight, incubated either with serum-free medium (for basal conditions) or with TM (0.01 μΜ and 0.05 μΜ) for 48 h in serum-free medium, and were proceeded for total RNA isolation. U251-MG cells were seeded in 6-well plates for 24 h and starved overnight at 60% confluency. Following starvation, cells were transfected with siRNA (siControl and siSRGN) particles as described previously. For RNA isolation, U251-MG cells were treated with siRNA for 48 h. Total RNA was isolated using NucleoSpin^®^ RNA kit (Macherey-Nagel GmbH & Co. KG, Dueren, Germany) following the manufacturer’s instructions. RNA concentration was determined by the absorbance measured at 260 nm. cDNA synthesis was performed using PrimerScript™ Reagent kit (Perfect Real Time PCR, Takara Bio, Göteborg, Sweden) according to manufacturer’s instructions. Real-Time qPCR analysis was conducted in 20 μL reaction mixture using the reaction mixture KAPA SYBR^®^ Fast qPCR kit Master Mix (2×) Universal (Kapa Biosystems, Sigma-Aldrich, St. Louis, MO, USA) according to manufacturer’s instructions. The amplification was performed utilizing Rotor Gene Q (Qiagen, Germantown, MD, USA) and relative quantification of the data was obtained using ΔΔCt method, with 18S rRNA gene being used as a normalizer. In addition, a melting curve analysis was always performed for detecting the SYBR Greenbased objective amplicon. Fold changes were determined as 2^−ΔΔCt^. Genes of interest and utilized primers are presented in Table S1.

### 2.3. Immunoblotting

LN-18 cell lines were seeded in 60 mm dishes and grown in complete medium up to 70–80% confluency. Cells were starved overnight, incubated either with serum-free medium (for basal conditions) or with TM (0.01 μΜ and 0.05 μΜ) for 48 h in serum-free medium. U251-MG cells were seeded in 6-well plates for 24 h and starved overnight at 60% confluency. Following starvation, cells were transfected with siRNA (siControl and siSRGN) particles as described previously. For protein isolation, U251-MG cells were treated with siRNA for 72 h. Following the respective treatment, attached cells were washed with cold PBS and lysed using scraper with Lysis Buffer: 25 mM HEPES, pH 7.5, 150 mM NaCl, 5 mM EDTA, 10% (*v*/*v*) glycerol, 1% (*v*/*v*) Triton X-100, containing protease inhibitor cocktail (#20-201 Millipore, Sigma-Aldrich, St. Louis, MO, USA) and 0.5 mM sodium orthovanadate (#S6508, Sigma-Aldrich, St. Louis, MO, USA). Samples were reduced with β-mercaptoethanol in Laemmli sample buffer, separated by SDS-PAGE in 10% poly-acrylamide gels and transferred to polyvinylidene difluoride (PVDF) membranes (Macherey-Nagel GmbH & Co. KG, Dueren, Germany). The membranes were blocked in 5% (*w*/*v*) Bovine Serum Albumin (BSA) (Sigma-Aldrich, St. Louis, MO, USA) in Tris-buffered saline pH 7.4, containing 0.1% Tween-20 (TBS/Tween) for 1.5 h at room temperature and were then incubated with primary antibodies overnight at 4 °C. After three washes in TBS/Tween, membranes were further incubated with peroxidase-conjugated secondary goat anti-rabbit IgG (#A0545, Sigma-Aldrich, St. Louis, MO, USA) or anti-mouse IgG (#A4416, Sigma-Aldrich, St. Louis, MO, USA) for 1.5 h at room temperature (TBS/Tween 1% BSA) and visualized by chemiluminescence (Luminata™ Crescendo Western HRP Substrate, Millipore, Sigma-Aldrich, St. Louis, MO, USA). The density of immunoreactive bands was quantified using Image J Fiji Software (https://imagej.net/software/fiji/). Primary antibodies used in immunoblotting analyses include: phospho-Akt (Cell Signaling Technology Inc., Danvers, MA, USA #9271, dilution 1:1000), total-Akt (Cell Signaling Technology Inc., #9272, dilution 1:1000), ATF4 (Cell Signaling Technology Inc., #11815, dilution 1:1000), ATF6 (Cell Signaling Technology Inc., #65880, dilution 1:1000), BiP (Cell Signaling Technology Inc., #3183, dilution 1:1000), caspase-3 (Cell Signaling Technology Inc., #9665, dilution 1:1000), CHOP (Cell Signaling Technology Inc., #2895, dilution 1:1000), phospho-eIF2α (Cell Signaling Technology Inc., #3398, dilution 1:1000), total-eIF2α (Cell Signaling Technology Inc., #5324, dilution 1:1000), phosphor-IRE1 (Novus Biologicals, Centennial, CO, USA, NB100-2323SS, 1:1000), total-IRE1 (Cell Signaling Technology Inc., #3294, dilution 1:1000), PARP-1 (abcam, Cambridge, UK, ab194586, dilution 1:1000), phospho-PERK (Cell Signaling Inc., #3179, dilution 1:1000), total-PERK (Cell Signaling Inc., #3192, dilution 1:1000), phospho-p65 (Santa Cruz Biotechnology, Inc., #sc-136548, dilution 1:750), total-p65 (Santa Cruz Biotechnology, Inc., #sc-8008, dilution 1:500), α-tubulin (Sigma-Aldrich, #T9026, dilution 1:4000) and β-actin (Santa Cruz Biotechnology, Inc., #sc-69879, dilution 1:2000).

### 2.4. Immunofluorescence/Phase-Contrast Microscopy

LN-18 cell lines were seeded on UV-exposed sterile glass coverslips in 24-well plates and grown in complete medium up to 60–70% confluency. Cells were starved overnight, incubated either with serum-free medium (for basal conditions) or with TM (0.01 μΜ and 0.05 μΜ) for 48 h in serum-free medium. Afterwards, cells were washed with PBS and fixed with 4% paraformaldehyde (PFA) in PBS, permeabilized with 0.05% Triton X-100 in PBS/0.01% Tween-20 (PBS/Tween) and blocked with 5% BSA in PBS/Tween solution. Coverslips were incubated with primary antibody against ATF4 (Cell Signaling Technology Inc., #11815, dilution 1:100) or phospho-p65 (Santa Cruz Biotechnology Inc., #sc-136548, dilution 1:100), overnight at 4 °C in chamber to maintain humidity. Following the primary antibody incubation, the secondary antibody Alexa Fluor 594 goat anti-mouse (Invitrogen, Thermo Fisher Scientific Inc., Waltham, MA, USA, A-11032, dilution 1:1000) or Alexa Fluor 594 goat anti-rabbit (Invitrogen, A-11012, dilution 1:1000) were used and the coverslips were mounted on microscope slides using mounting with DAPI. All washing steps between antibodies and DAPI incubation were conducted with PBS/Tween. Coverslips were observed with a fluorescent phase contrast microscope [OLYMPUS CKX41 with a color digital camera CMOS (SC30)] at 60× utilizing the Image Pro-Plus program (Media Cybrnetics, Inc., Rockville, MD, USA). Phase contrast images were also captured with the OLYMPUS CKX41 microscope at 10×.

### 2.5. Cathepsin B Zymography Assay

LN-18 cell lines were seeded in 60 mm dishes and grown in complete medium up to 70–80% confluency. Cells were starved overnight, incubated either with serum-free medium (for basal conditions) or with TM (0.01 μΜ and 0.05 μΜ) for 48 h in serum-free medium. Following treatment, attached cells were washed with cold PBS and lysed using scraper with lysis buffer. To avoid inhibiting cathepsin B cysteine activity, lysis buffer and protease inhibitors’ cocktail were prepared with the following: 25 mM HEPES, pH 7.5, 150 mM NaCl, 5 mM EDTA, 10% (*v*/*v*) glycerol, 1% (*v*/*v*) Triton X-100, ε-aminocaproic (2 mM), phenylmethylsulfonyl fluoride (PMSF, 1 mM) and benzamidine hydrochloride (1 mM). Equal amounts of protein extracts were incubated in Laemmli buffer without β-mercaptoethanol for 30 min at 37 °C. Proteins were separated by SDS-PAGE in 10% polyacrylamide gel supplemented with 2% (*w*/*v*) gelatin. After electrophoresis, gels were washed twice with 65 mM Tris-HCl pH = 7.4, 20% (*v*/*v*) glycerol buffer. Gels were washed with incubation buffer for permitting the enzymatic activity of intracellular lysosomal cathepsin B: 0.1 Μ sodium phosphate pH = 4, 1 mM EDTA, 2 mM DTT and incubated for 1 day at 37 °C. Buffer for gel staining included 0.25% (*w*/*v*) Coomassie Blue R-250, 43% methanol, 7% acetic acid and 50% distilled water. Gels were incubated with staining buffer for 30 min at room temperature and washed twice with distilled water. Two washes, 10 min each, were performed with 50% methanol, 10% acetic acid and 40% distilled water solution to destain gels. Finally, gels were washed and maintained in 7% acetic acid prior to image capturing. Areas of protease activity were detected as clear bands against the blue background and their density was determined by using Image J Software.

### 2.6. Proliferation Assay

LN-18 cell lines were seeded in 24-well plates and grown in serum-containing medium up to 70% confluency. Cells were starved overnight, incubated with TM (0.001, 0.01, 0.05, 0.1, 10, 100 and 200 μΜ) for 48 h in serum-free medium. U251-MG cells were seeded in 24-well plates for 24 h and starved overnight at 60% confluency. Following starvation, cells were transfected with siRNA (siControl and siSRGN) particles as described previously, for 48 h. For UPR inhibition protocol, LN-18 cell lines were seeded in 24-well plates and starved overnight upon 70–80% confluency. Cells were incubated with 0.1, 1 and 10 μΜ of the respective inhibitors for 24 h (MKC8866, Kira 6, GSK2606414 and Ceapin-A7). Each negative control contained the respective volume of DMSO within serum-free medium. Afterwards, cells were detached through trypsinization, centrifuged at 6000 rpm for 6 min, stained with trypan blue and counted directly using a hemocytometer. The detection of cell proliferation was also confirmed by conducting the Premix WST-1 Cell Proliferation Assay System (Takara Bio, Göteborg, Sweden) at a 1:10 ratio, according to the manufacturer’s protocol. Data were obtained by measuring the absorbance at 450 nm (reference wavelength at 650 nm).

### 2.7. Wound Healing Assay

LN-18 cell lines were seeded in 24-well plates and grown in serum-containing medium until confluent cell layers. Cells were starved overnight and wounded by scratching with a sterile 100 μL pipette tip. Detached cells were removed by washing with PBS and cells were incubated for 45 min at 37 °C with serum-free medium containing 10 μΜ of the cytostatic agent cytarabine (Sigma-Aldrich) and TM (0.01, 0.05 and 0.1 μΜ) for 48 h in serum-free medium. In all experimental conditions, images of the wound were captured after the 45 min incubation (0 h), in 24 h and 48 h of incubation. The wound closure was monitored using a digital camera connected to a microscope [OLYMPUS CKX41 microscope with a color digital camera CMOS (SC30)] and wound surface area was quantified using Image J Software.

### 2.8. Patient Cohort and Data Selection

Transcriptomic and clinical data were obtained from the TCGA glioblastoma cohort using the offline GloVis dataset. Only primary IDH-wt glioblastoma samples were included to match the biological context of the experimental models.

### 2.9. Gene Expression Processing

Gene expression values were analyzed on the log2-transformed expression scale provided in the dataset. *SRGN* expression was analyzed as the continuous variable. A composite UPR-inflammation transcriptional score was calculated from the following genes *EIF2AK3*, *ERN1*, *ATF6*, *HSPA5*, *EIF2S1*, *ATF4*, *DDIT3*, *XBP1*, *TLR2*, *TLR4*, *TNFRSF1B*. For each patient, the score was computed as the mean expression across all variables.

### 2.10. Correlation Analysis

Spearman’s rank correlation was applied to assess associations between *SRGN* expression and the UPR-inflammation score, as well as between individual genes. Adjustments for multiple comparisons were made, where appropriate, using the Benjamini-Hochberg method (FDR).

### 2.11. Survival Analysis

Kaplan-Meier survival curves were generated and compared using log-rank test and Gehan-Breslow-Wilcoxon test. Cox proportional hazards models were fitted to evaluate continuous effects. The proportional hazards assumption was tested using Schoenfeld residuals.

### 2.12. Statistical Analysis

For each assay, individual experiments were conducted at least 3 times. Data in diagrams are expressed as mean ± standard deviation (SD). Statistically significant differences were evaluated using an unpaired two-tailed *t*-test. Statistical analyses and graphs were made using GraphPad Prism 9 (GraphPad Software). Statistically significant differences are indicated by asterisks: * (*p* ≤ 0.05). Patients’ gene expression, correlation and survival analyses were performed in RStudio (version 2024.04.0+735, executed by Posit Software, PBC, version 2024.11) for MacOS.

## 3. Results

### 3.1. SRGN Suppression Abolishes UPR Markers’ Expression in GBM Cells, and Affects the Response to TM-Induced ER Stress

**Figure 1:**
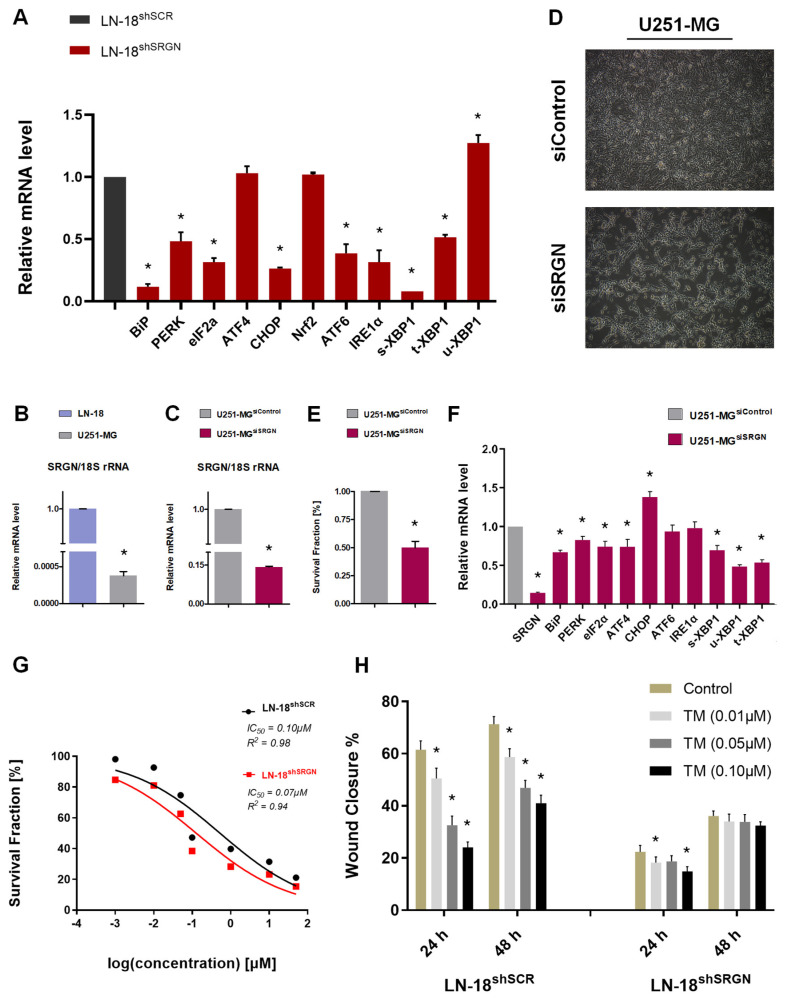
**Loss of SRGN diminishes the expression of UPR markers in GBM cell lines and results in ER stress susceptibility.** (**A**) Assessment of the mRNA levels of UPR mediators in LN-18^shSCR^ and LN-18^shSRGN^ cells through real-time qPCR. (**B**) Relative mRNA levels of *SRGN* in LN-18 and U251-MG cell lines. (**C**) *SRGN* suppression levels in U251-MG^siSRGN^ cells via real-time qPCR and (**D**) phase-contrast microscopy images upon *SRGN* silencing in U251-MG cells (48 h). (**E**) Relative proliferation rates of U251-MG^siControl^ and U251-MG^siSRGN^ cells after transient SRGN silencing (48 h). (**F**) Evaluation of the gene expression of UPR markers in U251-MG^siControl^ and U251-MG^siSRGN^ cells (48 h). (**G**) IC50 values for LN-18 cell lines upon TM treatment (48 h). (**H**) Wound closure assay in LN-18 cell lines after TM treatment for 24 h and 48 h. Statistically significant differences compared to control are shown by asterisk: * (*p* ≤ 0.05).

To evaluate the role of SRGN as a potential regulator of the UPR pathway, we first examined the gene expression of key UPR effectors (Figure 1A). Previous studies have indicated that PERK branch acts as a kinase of two substrates, eukaryotic translation initiation factor 2α (eIF2α or EIF2S1) and nuclear factor erythroid-derived-2-related-2 (Nrf2). The phosphorylation of the former leads to inactivation of eIF2α and concomitant transient inhibition of global protein synthesis for ER stress alleviation. On the other hand, Nrf2 phosphorylation activates the latter to induce the expression of antioxidant enzymes [44,45,46,47]. eIF2α inhibition leads indirectly to the preferential expression of ATF4 and C/EBP homologous protein (CHOP or DDIT3), active transcription factors (TFs) that regulate protein folding, autophagy and cell death/survival [48,49]. We found that loss of SRGN led to a 50% downregulation of *PERK* mRNA levels, accompanied by more than 60% reduction of both *eIF2α* and *CHOP* in LN-18^shSRGN^ cells. Similarly, *BiP* and *ATF6* transcription levels were attenuated by 90% and 60% respectively upon SRGN depletion (Figure 1A). It is well documented that IRE1 possesses both kinase and RNase activity. Uncoupling of BiP allows IRE1 to act as a kinase and undergo trans-autophosphorylation, without recognizing different substrates to phosphorylate. Phosphorylated IRE1 then exhibits its RNase activity to splice the mRNA of X-box binding protein 1 (XBP1), thus giving rise to a stable and active spliced s-XBP1 TF [38,50,51,52,53,54]. *IRE1* and pre-mature total t-*XBP1* mRNA levels were detected 60% and 50% reduced, and simultaneously s-*XBP1* was abolished in LN-18^shSRGN^ cells. On the contrary, non-modified unspliced u-*XBP1* was slightly upregulated in LN-18^shSRGN^ cells (Figure 1A). We expanded this protocol to another aggressive GBM cell line, U251-MG, which expresses significantly lower basal levels of *SRGN* compared to LN-18 cells (Figure 1B). After transient silencing of *SRGN* through siRNA, we observed that U251-MG^siSRGN^ cells were less proliferative, formed cell aggregates and displayed a differentiated and apoptotic phenotype (Figure 1C–E). The same trend observed in LN-18^shSRGN^ cells was replicated in U251-MG^siSRGN^ cells, with all UPR markers’ expression being moderately decreased, besides *CHOP*, upon *SRGN* silencing (Figure 1F). All the above pinpoint the fact that UPR mediators are affected by SRGN levels, suggesting that *SRGN* expression is essential for all three UPR branches.

Considering the significant reduction in the expression of UPR mediators in LN-18^shSRGN^ cells, we utilized TM to induce ER stress in both LN-18 cell lines. The IC50 values indicated that LN-18^shSRGN^ cells are slightly more sensitive to ER stress-induced cell death (Figure 1G), while migration was affected in both cell lines in a dose dependent manner (Figure 1H). Although TM decreased wound closure predominantly of LN-18^shSCR^, SRGN-expressing cells maintained a more potent migratory capacity compared to SRGN-suppressed, even upon extensive ER stress (Figure 1H).

### 3.2. Loss of SRGN Attenuates UPR Activation in GBM Cells

**Figure 2:**
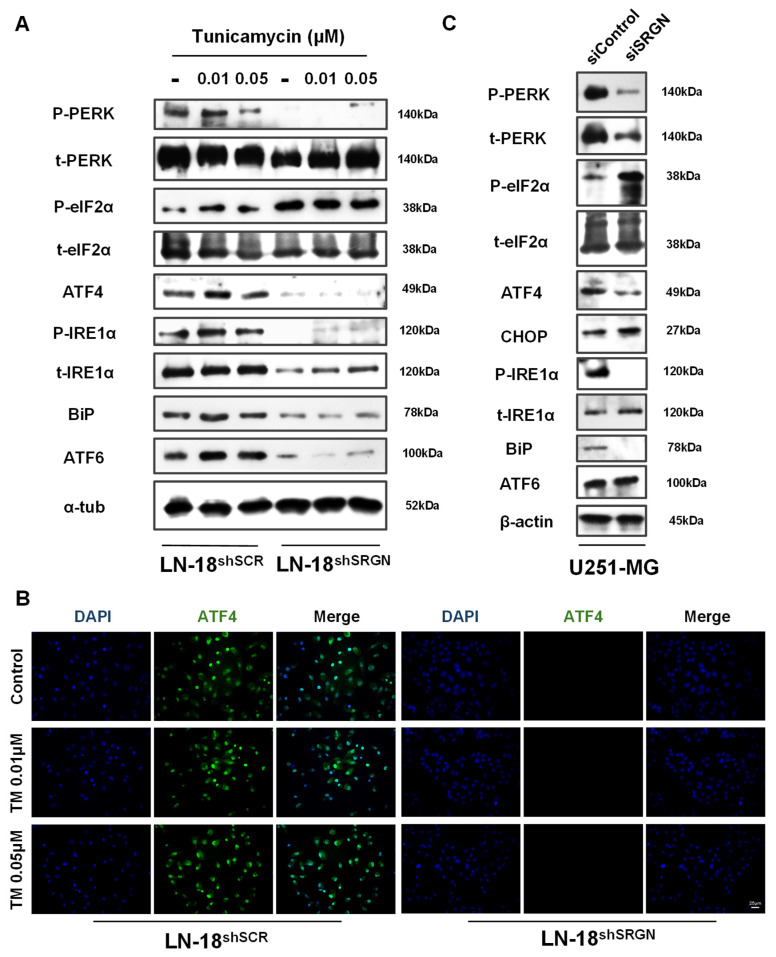
**SRGN depletion attenuates the activation profile of UPR in GBM cell lines and renders cells non-responsive to ER stress.** (**A**) Western blot analysis of UPR effectors’ protein and phosphorylated levels in TM-treated (48 h) LN-18 cells. (**B**) Immunofluorescence staining for ATF4 (green) and nuclei (blue) depicting the distribution and nuclear accumulation of ATF4 in TM-treated (48 h) LN-18^shSCR^ cells. ATF4 was not detected in LN-18^shSRGN^ cells under relative exposure. Scale bar 25 μm. (**C**) Western blot analysis of the basal UPR protein and phosphorylated levels in U251-MG^siControl^ and U251-MG^siSRGN^ cells, following transient SRGN silencing (72 h). All blots are representative of at least three independent experimental repetitions.

To determine whether mRNA levels’ alterations are reflected in the active status of UPR, we went on with the evaluation of the phosphorylation status and protein levels of UPR markers in TM treated LN-18 cells (Figure 2A). Firstly, we confirmed that SRGN depletion in LN-18^shSRGN^ cells diminished both mRNA and protein levels of all examined UPR markers, and interestingly ATF4 protein expression was also downregulated (Figure 2A), in contrast to its gene expression (Figure 1A). Simultaneously, the active phosphorylated levels of both PERK and IRE1 were also diminished in LN-18^shSRGN^ cells (Figure 2A). eIF2α was found heavily phosphorylated in LN-18^shSRGN^ cells, suggesting that PERK inactivation inhibits protein synthesis in less aggressive SRGN-suppressed cells. Similarly, both BiP and ATF6 expression was found to be 60% lower in LN-18^shSRGN^ cells (Figure 2A). Furthermore, long-term (48 h) treatment with 0.01 μM TM revealed that LN-18^shSCR^ cells probably adapt to prolonged but mild ER stress by globally inducing the levels and/or activation of UPR mediators, whereas excessive ER stress (0.05 μM TM) reversed this upregulation. In contrast with the above, TM treatment did not significantly affect neither the expression nor the activity of UPR in LN-18^shSRGN^ cells, which were overall non-responsive to ER stress induction (Figure 2A). Of importance was the finding that ATF4 was constitutively accumulated within the cell nuclei of LN-18^shSCR^ cells, even in the absence of TM, and was not detected in LN-18^shSRGN^ cells (Figure 2B). ER stress induction did not affect its localization, while under basal conditions ATF4 appeared to exhibit its activity as a TF, suggesting that SRGN-expressing LN-18^shSCR^ cells display a constitutively active UPR system.

The examination of the basal UPR markers’ levels and activity in U251-MG cells also confirmed that even transient *SRGN* knock-down resulted in significant downregulation of UPR effectors in U251-MG^siSRGN^ cells (Figure 2C). PERK expression and activation were hindered upon *SRGN* silencing, together with the active form of IRE1 in U251-MG^siSRGN^ cells, while no differences were detected regarding ATF6. ATF4 and BiP protein levels were also decreased, accompanied by apparent phosphorylation and inhibition of eIF2α in SRGN-suppressed U251-MG^siSRGN^ cells, resembling the behavior of LN-18^shSRGN^ cells (Figure 2A,C). Contradictory, *CHOP* experienced upregulation in both gene and protein expression in U251-MG cells (Figure 1F and Figure 2C), indicating distinct roles within different GBM cell lines, as noticed in different studies [55,56,57]. Nevertheless, these results propose that SRGN possesses a global role in regulating UPR basal levels, activity and potential in GBM cells.

### 3.3. SRGN Maintains an Active Pro-Survival UPR Pathway in GBM Cells

**Figure 3:**
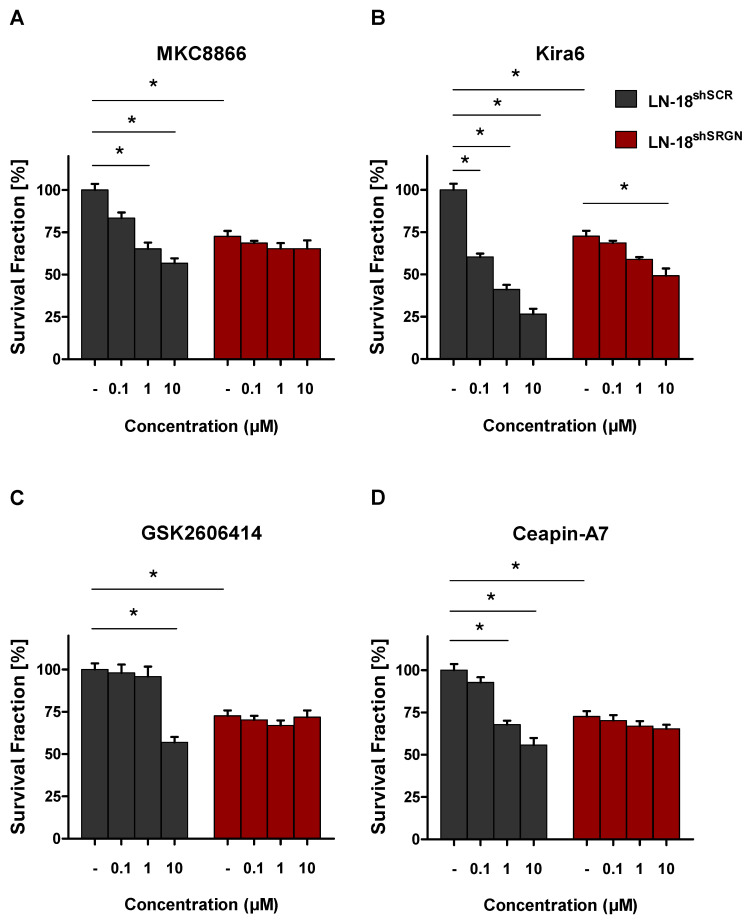
**SRGN-expressing cells possess a pro-survival UPR mechanism and demand global UPR activation.** Assessment of LN-18^shSCR^ and LN-18^shSRGN^ cells’ viability upon treatment with (**A**) MKC8866 (IRE1 RNase activity inhibitor), (**B**) Kira6 (IRE1 kinase activity inhibitor), (**C**) GSK2606414 (PERK kinase activity inhibitor) and (**D**) Ceapin-A7 (ATF6 TF activity inhibitor) for 24 h incubation at 0.1, 1 and 10 μΜ final concentrations. Statistically significant differences compared to control are shown by bars and asterisk: * (*p* ≤ 0.05).

The dual role of UPR in reinstating the cell death/survival equilibrium is a largely unidentified topic in cancer biology. There are numerous contradicting studies showing that UPR markers, even within the same pathologies, can emerge as apoptosis inhibitors or apoptotic mediators, depending on slight perturbations, ER stress intensity and sensitive distinctions on upstream stimuli [58,59,60]. Our aim was to understand the setting upon which SRGN regulates all three UPR branches, as well as their interconnection, to determine cell fate. To this end, we utilized UPR inhibitors to suppress IRE1 RNase activity (MKC8866), IRE1 kinase activity (Kira 6), PERK kinase activity (GSK2606414) and ATF6 TF activity (Ceapin-A7). Treatment with the above confirmed that inhibiting each UPR branch alone resulted in loss of LN-18^shSCR^ cells’ viability in a dose-dependent manner, whereas LN-18^shSRGN^ cells were unaffected (Figure 3A–D). Of interest was the finding that, regarding cell proliferation, disruption of IRE1 kinase activity was more effective than IRE1 RNase activity suppression (Figure 3A,B), and that Kira 6 was the most potent inhibitor among all (Figure 3A–D). These observations suggest that the phosphorylation status of IRE1 is also consequential for cell homeostasis independently of XBP-1 function, as well as that IRE1 might be the most prominent UPR sensor for GBM cells’ fidelity. Considering this data, we conclude that SRGN-expressing LN-18^shSCR^ cells possess a pro-survival UPR and utilize all three branches to maintain their oncogenic properties.

### 3.4. SRGN Depletion Provokes an Apoptotic Phenotype in GBM Cells

**Figure 4:**
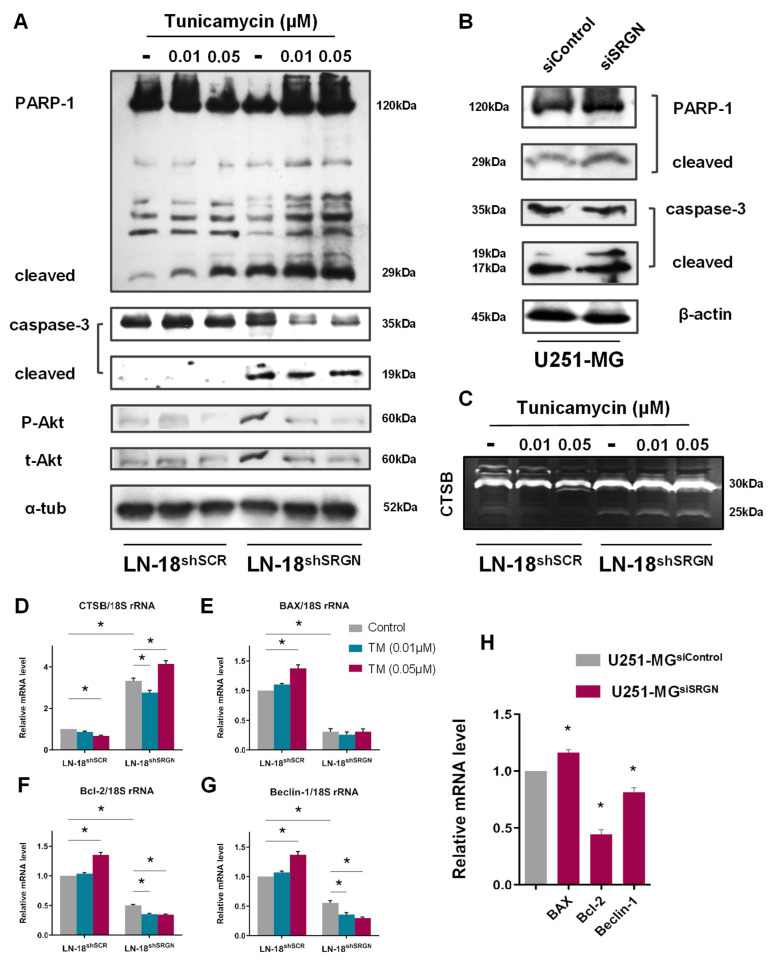
**SRGN suppression provokes an apoptotic phenotype in GBM cells.** (**A**) Western blot analysis of the intact and cleaved forms of caspase-3 and PARP-1, as well as the active and total Akt forms in TM-treated (48 h) LN-18 cells. (**B**) caspase-3 and PARP-1 proteolysis assessed through Western blotting in U251-MG cells after transient SRGN knock-down (72 h). (**C**) Zymography assay for determining the active intracellular CTSB isoforms in acidic pH upon TM treatment (48 h) in LN-18 cell lines. Real-time qPCR for evaluating the relative mRNA levels of (**D**) *CTSB*, (**E**) *BAX*, (**F**) *Bcl-2* and (**G**) *Beclin-1* in TM-treated (48 h) LN-18 cell lines. (**H**) Basal mRNA levels of *BAX*, *Bcl-2* and *Beclin-1* in U251-MG cells upon SRGN depletion (48 h). All blots and zymography images are representative of at least three independent experimental repetitions. Statistically significant differences compared to control are shown by bars and asterisk: * (*p* ≤ 0.05).

Consistently with LN-18 cells, loss of SRGN in U251-MG cells correlated with loss of proliferation capacity and induced apoptosis of U251-MG^siSRGN^ cells (Figure 1D). Additionally, ER stress induction highlighted a differential response of LN-18 cells regarding UPR activity and cell viability (Figure 1G and Figure 2A). To decipher the potential apoptotic mechanisms that result in LN-18^shSRGN^ cell death, we evaluated the cleavage of the pro-apoptotic marker caspase-3 and its substrate’s cleavage, poly-ADP-ribose polymerase 1 (PARP-1), in TM-treated LN-18 cells (Figure 4A). We have already shown that Akt is paradoxically overexpressed in LN-18^shSRGN^ cells [20]. We found that caspase-3 is constitutively cleaved and active in SRGN-suppressed LN-18^shSRGN^ cells, whereas only the intact form was detected in control SRGN-expressing LN-18^shSCR^ cells (Figure 4A). Even though TM did not significantly affect Akt phosphorylation in LN-18^shSCR^ cells, ER stress induction led to apparent decrease of its activity in LN-18^shSRGN^ cells (Figure 4A). Treatment with TM pointed out that PARP-1 was cleaved in a dose-dependent manner in both LN-18 cell lines, but LN-18^shSCR^ displayed resistance to ER stress-induced apoptosis compared to LN-18^shSRGN^ cells (Figure 4A). Following caspase-3 activity, LN-18^shSRGN^ cells degraded PARP-1 at basal levels, as shown by the 29 kDa fragment and, to a lesser extent, by the 89 kDa fragment (Figure 4A). *SRGN* silencing also provoked caspase-3 and PARP-1 cleavage in U251-MG^siSRGN^ cells, while the extensive PARP-1 degradation observed in LN-18 was not present in U251-MG cells (Figure 4B). Based on the above we suggest that SRGN depletion stimulates the caspase-dependent apoptotic cascade and sensitizes GBM cells to ER stress-associated cell death.

The extensive degradation of PARP-1 in LN-18 cells, the low levels of the 89 kDa PARP-1 fragment, as well as the absence of cleaved caspase-3 in LN-18^shSCR^ cells (Figure 3A), led to the inquiry of distinct proteases that cleave PARP-1. Literature points out that cathepsins (CTSs) and specifically lysosomal intracellular CTSB recognizes PARP-1 and simulates caspase-3 as a proteolytic enzyme catalyzing cell death in a context-dependent manner [61]. To evaluate the levels of CTSB, we performed zymography of intracellular CTSB from lysates of TM-treated LN-18 cells. The enzymatic activity of both 25 kDa and 30 kDa CTSB isoforms was found to be present at basal levels in LN-18^shSCR^ cells, whereas they were enhanced upon TM treatment in a dose-dependent manner in LN-18^shSRGN^ cells (Figure 4C). *CTSB* mRNA levels were correlated with its activity, while intriguingly TM reduced both its mRNA and activity levels in LN-18^shSCR^ cells (Figure 4C,D).

Further analysis of the mitochondrial pro-apoptotic and anti-apoptotic markers Bcl-2-associated X protein (*BAX*) and B-cell leukemia 2 (*Bcl-2*), respectively, showed that both were more than 60% downregulated in LN-18^shSRGN^ cells (Figure 4E,F). TM treatment at high concentrations led to an increase of both *BAX*/*Bcl-2* in LN-18^shSCR^ cells, while a similar trend was observed at the mRNA levels of apoptosis and autophagy regulator *Beclin-1* (Figure 4E–G). The anti-apoptotic markers *Bcl-2* and *Beclin-1* were downregulated in SRGN-suppressed LN-18^shSRGN^ cells upon TM treatment (Figure 4F,G). Similarly, transient silencing of *SRGN* in U251-MG^siSRGN^ cells resulted in apparent downregulation of *Bcl-2* (50%), a mild decrease in *Beclin-1* expression, and an induction of *BAX* expression, accompanied by elevated caspase-3 activation and PARP-1 cleavage (Figure 4B,H). Collectively, SRGN potentially inhibits the apoptotic cascade via diverse pathways and provides resistance to ER stress and concomitant cell death.

### 3.5. SRGN Participates in a Pro-Survival Inflammatory Response in GBM Cells

**Figure 5:**
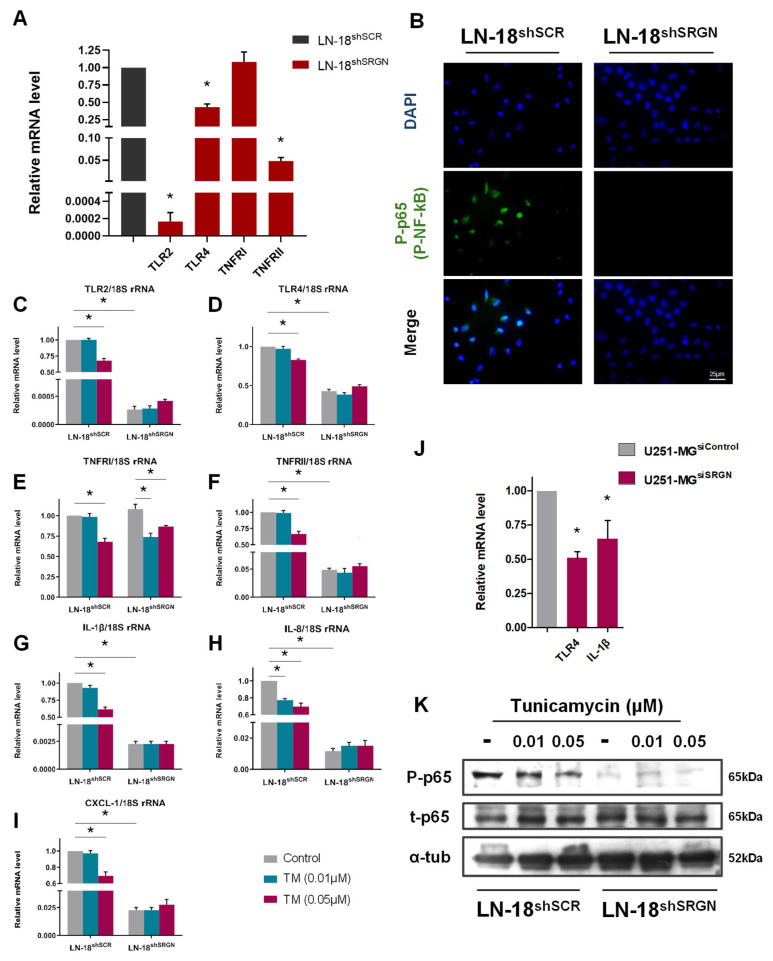
**SRGN orchestrates the inflammatory response in GBM cells.** (**A**) Relative mRNA levels of TLRs and TNFRs in LN-18^shSCR^ and LN-18^shSRGN^ cell lines assessed through real-time PCR. (**B**) Immunofluorescence staining for P-p65 (green) and nuclei (blue) depicting the distribution and nuclear accumulation of phosphorylated p65 subunit in LN-18 cells at basal conditions. P-p65 was not detected in LN-18^shSRGN^ cells under relative exposure. Scale bar 25 μm. Real-time qPCR for evaluating the relative mRNA levels of (**C**) *TLR2*, (**D**) *TLR4*, (**E**) *TNFRI*, (**F**) *TNFRII*, (**G**) *IL-1β*, (**H**) *IL-8* and (**I**) *CXCL-1* in TM-treated (48 h) LN-18 cell lines. (**J**) Basal mRNA levels of *TLR4* and *IL-1β* in U251-MG cells upon transient *SRGN* silencing (48 h) assessed through real-time qPCR. (**K**) p65/NF-kB phosphorylated and total forms in TM-treated (48 h) LN-18 cell lines evaluated by Western blotting. All blots are representative of at least three independent experimental repetitions. Statistically significant differences compared to control are shown by bars and asterisk: * (*p* ≤ 0.05).

To expand on the context of cellular homeostasis, we assessed the levels and activity of inflammatory mediators associated with the UPR pathways. There is extensive literature describing the roles of toll-like receptors (TRLs) as upstream regulators of UPR independently of ER stress, especially in MM cells [62,63]. Consequently, nuclear factor-kappa B (NF-kB) and its’ inhibitor (IKKα/β) are modulated to substantiate signal transduction irrespective or depending on UPR activity [64]. Interleukins (ILs) and other chemokines, such as the CXC motif chemokine ligand 1 (CXCL-1) belong to the gene-targets induced by inflammatory responses or can function as ligands initiating the above phenomena. GBM cells utilize these signaling cascades to advance pro-survival efforts and tumorigenicity, while it is also well-documented that UPR pathway can oversee inflammation and vice versa [65,66]. Thus, we performed analysis to quantify the relative gene expression of TLRs and tumor necrosis factor receptors (TNFRs) in LN-18 cells. *TLR2* expression was abolished upon SRGN depletion, together with a 60% and 95% reduction of *TLR4* and *TNFRII* (*TNFRSF1B*), respectively, in LN-18^shSRGN^ cells, while *TNFRI* was not affected (Figure 5A). These significant alterations were reflected in the active form of NF-kB, the phosphorylated p65 subunit, which was constitutively nuclear in LN-18^shSCR^ cells and not detected in LN-18^shSRGN^ cells (Figure 5B).

To decipher the response of LN-18 cells upon induced ER stress regarding the inflammatory-related signaling, we evaluated the effect of TM in the expression of the above key effectors. We found that LN-18^shSCR^ cells could adapt to mild ER stress (0.01 μM TM) without compensating the transcription rates of TLRs and TNFRs, whereas excessive ER stress downregulated their mRNA levels approximately around 30%. On the contrary, LN-18^shSRGN^ cells were non-responsive to TM and maintained the attenuated mRNA levels of the analyzed markers (Figure 5C–F). It is important to note that *TNFRI* is implicated in both cell death and survival, being significantly reduced even at low TM concentrations in LN-18^shSRGN^ cells (Figure 4E), while in contrast *TNFRII* predominantly stimulates pro-survival cues [67,68,69,70,71]. Alongside these, we observed a similar expression profile in *IL-1β*, *IL-8* and *CXCL-1*, which displayed a dose-dependent reduction upon TM treatment in LN-18^shSCR^ cells (Figure 5G–I). *SRGN* silencing also resulted in 50% downregulation of both *TLR4* and *IL-1β* mRNA levels in U251-MG^siSRGN^ cells (Figure 5J). Lastly, TM treatment attenuated NF-kB activity in a dose-dependent manner in LN-18^shSCR^ cells, suggesting that it could undergo, at least in part, receptor-dependent regulation (Figure 5K). Altogether, our results propose that SRGN-expressing GBM cells synchronize signaling networks to exhibit oncogenic and pro-survival inflammatory signals, resist and adapt to hostile conditions.

### 3.6. SRGN Correlated with a Coordinated UPR-Inflammation Transcriptional Program

**Figure 6:**
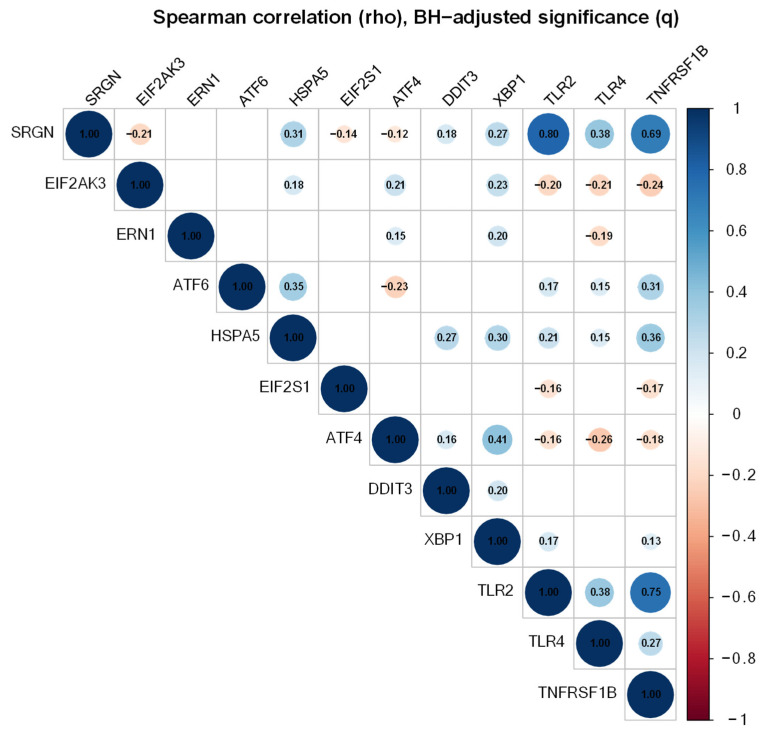
**Correlation matrix of SRGN and UPR–inflammation signature genes in primary IDH-wt glioblastoma.** Spearman correlation coefficients (rho) were calculated between *SRGN* and canonical unfolded protein response and inflammatory genes (*EIF2AK3*, *ERN1*, *ATF6*, *HSPA5*, *EIF2S1*, *ATF4*, *DDIT3*, *XBP1*, *TLR2*, *TLR4*, *TNFRSF1B*) within the TCGA GBM cohort filtered for primary IDH-wt tumours. Circle size and colour intensity represent the magnitude and direction of the correlation. Only statistically significant correlations after Benjamini–Hochberg multiple-testing correction (q < 0.05) are displayed; non-significant comparisons are omitted.

To evaluate whether the SRGN-UPR relationship observed in vitro is reflected at the transcriptomic and clinical level, the TCGA_GPM cohort was analyzed with the following clinical criteria primary tumor, IDH-wt glioblastoma. The composite UPR-inflammation score was constructed from canonical UPR components and inflammatory mediators (*EIF2AK3*, *ERN1*, *ATF6*, *HSPA5*, *EIF2S1*, *ATF4*, *DDIT3*, *XBP1*, *TLR2*, *TLR4*, *TNFRSF1B*). *SRGN* exhibited a strong positive association with the UPR-inflammation score (Spearman rho = 0.669; *q* = 9.7 × 10^−48^). Gene-wise analyses indicated that *SRGN* aligned more strongly with inflammatory mediators (*TLR2*, *TNFRSF1B*, *TLR4*) and selected UPR-linked transcripts (*HSPA5*, *XBP1*, *DDIT3*), whereas *ERN1* and *ATF6* showed no significant association after multiple-testing correction (Figure 6). These data support a coordinated SRGN-linked stress/inflammatory transcriptional program in primary IDH-wt GBM.

### 3.7. SRGN and UPR-Inflammation Define Distinct Survival Phenotypes in GBM

**Figure 7:**
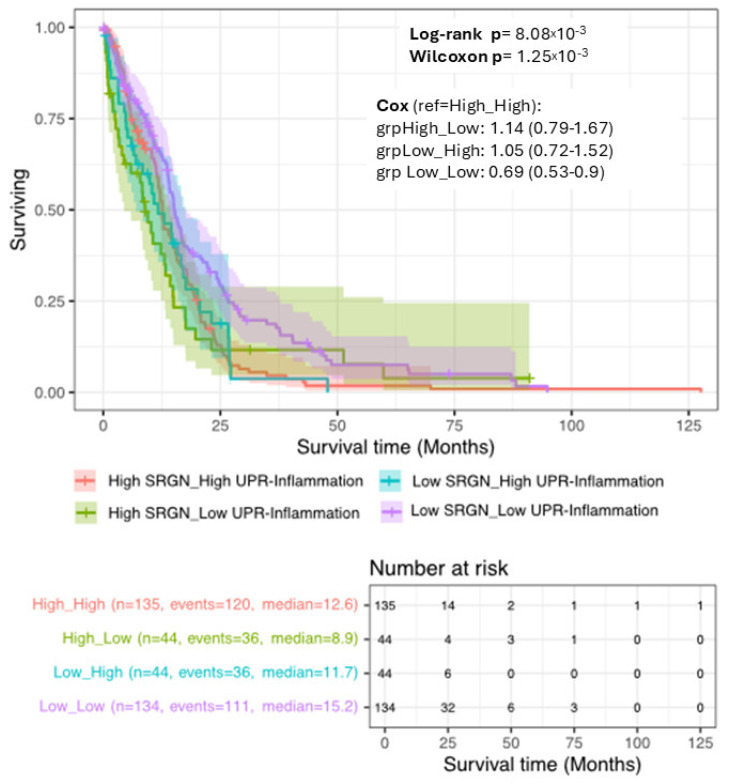
**Kaplan–Meier survival stratified by *SRGN* expression and UPR–inflammation activity.** Patients were divided into four groups using median cut-offs for *SRGN* expression and the UPR–inflammation score. Survival differed across groups (log-rank *p* = 8.08 × 10^−3^; Wilcoxon *p* = 1.25 × 10^−3^). The Low-*SRGN*/Low-UPR group showed the longest survival, whereas High-*SRGN*/Low-UPR showed the shortest. Median survival times (months): Low/Low 15.2, High/High 12.6, Low/High 11.7, High/Low 8.9. Curves are presented descriptively because proportional hazards assumptions were not satisfied.

Overall survival was examined using Kaplan-Meier estimates after stratifying patients into four groups based on median splits of *SRGN* expression and UPR-inflammation score (High/Low for each variable). Survival curves showed clear separation across the four strata, indicating heterogeneity in outcome according to the combined transcriptional state (Figure 7). Patients in the Low *SRGN*/Low UPR-inflammation group exhibited the most favourable survival profile with the longest median overall survival (15.2 months). In contrast, the High *SRGN*/Low UPR-inflammation group showed the poorest outcome, with the shortest median survival (8.9 months). The High *SRGN*/High UPR-inflammation and Low *SRGN*/High UPR-inflammation groups displayed intermediate survival trajectories, with median survivals of 12.6 and 11.7, respectively (Figure 7). The global separation of the curves was statistically significant by both the long-rank test (*p* = 8.08 × 10^−3^) and the Wilcoxon test (*p* = 1.25 × 10^−3^), indicating differences in survival distribution across groups, including at earlier time points. Visually, the curves suggest that combined low *SRGN* expression and low UPR-inflammation activity are associated with prolonged survival (Figure 7). However, given violations of the proportional hazards assumption observed in the subsequent Cox models, these Kaplan-Meier curves are presented descriptively, emphasizing differences in survival patterns rather than time-invariant hazard ratios.

## 4. Discussion

It is already well-established that SRGN is an oncogenic driver in many malignant tumors, while aspects of its mechanism of function remain to be elucidated. SRGN has been shown to induce numerous signaling cascades, among others the mitogen-activated protein kinases (MAPK) pathway, Src kinases, and the inflammatory response, predominantly through CD44 receptor [17,20]. In this study we investigated the versatile role of SRGN in regulating the UPR pathway, as well as its’ implication in inflammation, cell death and survival of GBM cells. Therefore, we utilized two aggressive GBM cell lines, LN-18 and U251-MG, expressing high levels of UPR markers. We developed stably transduced control LN-18^shSCR^ cells, also expressing high levels of SRGN, and SRGN-suppressed LN-18^shSRGN^ cells. U251-MG cells possess low basal levels of SRGN in comparison to LN-18 cells; thus, we performed transient silencing of *SRGN* through siRNA for each experimental procedure. Our first observation concerned the significant downregulation of UPR markers’ mRNA levels in SRGN-suppressed LN-18^shSRGN^ and U251-MG^siSRGN^ cells.

Taking into consideration the fact that UPR can exhibit a dual role in cell fate, with each effector displaying either apoptotic or pro-survival functions, we could not conclude how these alterations were associated with tumor progression [58,59]. Treatment with TM pinpointed that LN-18^shSRGN^ cells were more sensitive to ER stress induction and concomitant cell death, implying that decrease in UPR mediators’ expression was impactful in ER stress response. We noticed that LN-18^shSCR^ cells, even when experiencing excessive ER stress and proteotoxicity, maintained their oncogenic properties and expression of key tumorigenic factors, even higher than the basal levels of less aggressive LN-18^shSRGN^ cells, which were non-responsive to ER stress.

The apparent attenuation of UPR activity in SRGN-suppressed cells enhanced the notion that SRGN is a novel regulator of the UPR pathway. Simultaneously, the fact that ATF4 is localized within the cell nuclei of LN-18^shSCR^ cells under basal conditions, as well as the mild induction in UPR activity upon ER stress, suggest that SRGN-expressing LN-18^shSCR^ cells acquire a constitutively hyperactive UPR, probably to satisfy the extreme demands for protein synthesis and uncontrolled proliferation. Interestingly, SRGN was implicated in ATF4 expression only at the level of translation, while ATF4 gene expression and localization were not affected by the loss of SRGN. In agreement with the above, LN-18^shSCR^ cells retain the phosphorylated eIF2α in a relaxed state, while expressing it at high rates, providing an adaptable ‘switch’ of protein synthesis, in contrast to LN-18^shSRGN^ cells that undergo significant suppression of eIF2a activity. This phenomenon is widely observed in MM and early-stage transformation towards malignant myeloma clones, upon which hyperactive UPR depresses ER stress and cell death [72,73,74,75,76,77], while LN-18 and U251-MG GBM cells could resemble similar mechanisms.

Mechanistically, the PERK pathway could undergo auto-regulation by inducing the expression of both CHOP and growth arrest and DNA damage-inducible protein 34 (GADD34), thus controlling mitochondrial and caspase-dependent cell death [78], and hydrolyzing the phosphorylated eIF2α to restore its activity [79]. Our findings indicate that eIF2α could remain constitutively active to promote oncogenic protein synthesis in SRGN-expressing cells. In parallel, the protein and active levels of IRE1 were diminished in LN-18^shSRGN^ cells, together with the active form of IRE1 in U251-MG^siSRGN^ cells. Consequently, XBP1 splicing was abolished, while *XBP1* gene expression was also inhibited in SRGN-suppressed cells. These imply that SRGN-dependent XBP1 regulation takes place both at the level of gene expression and modification by IRE1 RNase activity. There is evidence pointing out that ATF4, ATF6, XBP1 and BiP are trans-activated through heterodimeric TF activity and bilateral gene expression induction, while u-XBP1 suppresses s-XBP1 function, a trend observed in LN-18 cells at the mRNA level [80,81,82]. Overall, we suggest that SRGN orchestrates the UPR transcription program and activity, while simultaneously UPR interplay reinforces UPR signaling upon a trans-activating positive feedback loop.

The implication of IRE1 in the regulated IRE1-dependent mRNA decay (RIDD), for ubiquitous protein synthesis inhibition and ER stress alleviation [83] was not evaluated in the present study, even though IRE1 attenuation in LN-18^shSRGN^ cells could also affect IRE1-dependent recycling mechanism. Nevertheless, we focused on IRE1 phosphorylation pattern as a cue for initiating or integrating inflammatory signaling. Tumor necrosis factor receptor-associated factor 2 (TRAF2) anchors upon phosphorylated IRE1, leading to the successive activation of apoptosis-signal-regulating kinase 1 (ASK1) and c-Jun N-terminal kinase (JNK). JNK and MAPK, as well as NF-kB and IKKα/β then execute an IRE1-dependent cascade to tightly adjust the apoptotic pathway collaterally with the UPR machinery [84,85,86]. This phenomenon can also occur in a ligand-independent manner, upon which phosphorylated IRE1 interacts and binds to TNFRI, to manipulate the expression of BAX, Bcl-2, Bcl-2 antagonist/killer protein (BAK), and Bcl-2 interacting mediator of cell death (BIM), thus abrogating the impact of stress-inducing apoptotic signals [87,88]. The perplex role of IRE1, especially in the context of receptor-dependent but ligand-independent pathway, provides important information about the constitutively hyperactive state of IRE1 even in the absence of extracellular and/or extrinsic stimuli, that could also partially underpin its function in SRGN-expressing GBM cells.

To decipher whether the UPR pathways of LN-18 cells correspond to integrated or terminal apoptotic UPR signaling, we inhibited each UPR branch alone. Our observation was that LN-18^shSCR^ cells experienced loss of viability upon treatment with each individual UPR inhibitor, whereas LN-18^shSRGN^ cells remained unaffected. We concluded that SRGN-expressing cells acquire a pro-survival UPR mechanism, confirming our hypothesis that SRGN maintains potent UPR activity in LN-18^shSCR^ cells for adapting to ER stress, while loss of SRGN and concomitant UPR impairment resulted in diminished tumorigenicity and cell death. This also explains the finding that UPR inhibitors were insufficient to provoke apoptosis in LN-18^shSRGN^ cells, given that they display attenuated UPR activation and do not fully utilize these pathways for their homeostasis. Additionally, we noticed that IRE1 RNAse activity inhibition, as well as kinase activity suppression, compromised LN-18^shSCR^ cells’ viability, but the latter was more effective. This implies that IRE1 mediates pro-survival cues via both XBP-1 and simultaneously through IRE1-dependent phosphorylation, but XBP1-independent cascades in SRGN-expressing cells. Lastly, IRE1 emerged as probably the most essential UPR sensor, considering that inhibition of its kinase activity, and inevitably downstream RNase activity, led to apparent cell death compared to inhibition of the other branches.

Alongside these data, caspase-3/PARP-1 activation upon TM treatment elicited the sensitization of LN-18^shSRGN^ cells to cell death due to UPR exhaustion. The moderate cleavage of PARP-1 in LN-18^shSCR^ cells, compared to the excessive apoptotic behavior of LN-18^shSRGN^ and U251-MG^siSRGN^ cells, signified that SRGN-depleted cells possess a ‘threshold’ in their oncogenic capacity. The attenuation of Akt activity in TM-treated LN-18^shSRGN^ cells also demonstrated the inability of SRGN-suppressed cells to maintain their cell viability. Concurrently, different studies have elaborated on the executionary part of UPR in cell death inhibition; both PERK/eIF2α/ATF4/CHOP and IRE1/XBP1 pathways induce Bcl-2 expression to suppress caspase-dependent apoptosis [87,89], while BiP non-canonical function also maintains low BAX/Bcl-2 expression ratios, provoking overall pro-survival signals in GBM [90]. This expression pattern was observed in LN-18 and U251-MG SRGN-expressing cells. The dual role of CHOP in determining cell death or survival was also apparent in this study. Herein, CHOP, BAX and caspase-3 stimulation in U251-MG^siSRGN^ cells could underscore a pro-apoptotic signal, as described in different studies [57], upon which CHOP acts as a positive regulator of BAX and concomitantly caspase-3. Altogether, our data encourage the presumption that loss of SRGN provokes ER stress-related cell death facilitated by caspase-dependent apoptosis, following UPR impairment.

The multiple fragments observed in PARP-1 blot signify that distinct proteases cleave PARP-1, especially in LN-18 cells. The characterization of its degradation products with different molecular weights has shown that lysosomal CTSs, mainly CTSB and CTSD, recognize PARP-1 as a substrate, and regulate autophagy and apoptotic events in a context-dependent manner [91]. This data could explain the correlation between excessive PARP-1 cleavage and CTSB levels in SRGN-suppressed LN-18^shSRGN^ cells. CTSB has been shown to act as an inhibitor of autophagy, regulating autophagosomes formation, and inducing apoptosis within cancer cells, by participating in inflammatory responses and consequential programmed cell death [92,93], arising further questions regarding its function upon SRGN depletion. The expression profile of Bcl-2, BAX and Beclin-1, further highlighted the disrupted adaptive mechanisms of SRGN-suppressed cells. Conclusively, there is adequate evidence that SRGN-expressing GBM cells acquire UPR-associated interconnected pathways to substantiate pro-survival and anti-apoptotic signaling, while the lysosomal-dependent autophagic flux and cellular recycling systems remain to be illuminated.

Studies of our group have shown that SRGN-expressing LN-18^shSCR^ cells express and secrete high levels of ILs, proposing that the inflammatory response is a tumorigenic factor in LN-18 cells [20]. In agreement with the above, receptors associated with UPR and inflammation were also found abrogated in LN-18^shSRGN^ and U251-MG^siSRGN^ cells, including TLR2/4, TNFRII and ILs. TLR4 has been highlighted as an inhibitor of eIF2α/ATF4/CHOP pathway of the apoptotic URP in H929 and JJN3 MM cells [94], whereas in the setting of a pro-survival UPR, TLRs have emerged as positive regulators of PERK and IRE1 branches [62,63]. Similarly, multiple studies have demonstrated that TNFRs stimulate IRE1 phosphorylation and XBP1-independent inflammation, inducing NF-kB signaling and ILs expression [69]. We also pinpointed the effect of intense ER stress in LN-18 cells, indicating that although LN-18^shSCR^ cells experienced moderate perturbations upon TM treatment and decreased their inflammatory signaling, LN-18^shSRGN^ cells were overall non-responsive and maintained abolished levels of TLRs, TNFRs, ILs, and phosphorylated NF-kB. Our results illustrate a strong correlation between the expression and activity of inflammatory mediators and UPR markers, suggesting that these two mechanisms communicate probably in a receptor-dependent manner.

The significance of the relationship between SRGN/UPR/inflammation in GBM was evaluated at the transcriptomic and clinical level, which revealed that *SRGN* is found to be co-expressed predominantly with the inflammatory receptors *TLR2/4* and *TNFRII*, as well as with *BiP*, *XBP1* and *CHOP* in GBM patients. Additionally, *TLR2* demonstrated positive correlation with both *TLR4* and especially *TNFRII*, implying the potent inflammatory signaling capacity of tumor cells. Kaplan-Meier curves provided important insights into the survival rates of GBM patients with simultaneously low expression of *SRGN*, UPR and inflammatory markers, being associated with better prognosis and prolonged life expectancy. On the contrary, high *SRGN* expression was found to be the crucial marker that hindered the survival time of GBM patients.

Overall, our results highlight SRGN as a global mediator of UPR activity, orchestrating a complex oncogenic network to manifest ER stress resistance, adaptation and survival of GBM cells. It is worth noting that UPR suppression has been in the focus of recent research, with MCK8866 and other blood-brain-barrier (BBB) permeable IRE1 and PERK inhibitors, displaying significant results in GBM treatment, predominantly by inducing sensitization to radiotherapy [89,95,96]. The hypoxic conditions of GBM TME, as well as the role of SRGN in ECM remodeling could also support the investigation of 5RN6-based carbonic anhydrase inhibitors in the setting of therapeutic approaches against GBM. UPR also operates in synchronization with the ubiquitin-proteasome system (UPS), that possesses significant roles in global protein recycling and cell signaling regulation. MM patients undergo treatment with bortezomib, an FDA-approved proteasome inhibitor, to suppress UPS-dependent degradation, leading to severe proteotoxicity and cell death. Independent studies have described the interplay between UPS and UPR, especially in MM cells, providing evidence that these systems exhibit bilateral regulation, while also participating in autophagic flux and the inflammatory response [97,98]. Targeting UPS through proteasome inhibitors, such as bortezomib and marizomib, accompanied by treatment with standard chemotherapeutic drugs, has reached the clinical levels in the effort of battling GBM, conferring the importance and clinical relevance of UPS as a homeostatic mechanism in aggressive malignancies [99,100,101,102,103,104,105].

Herein, these results do not demonstrate the exact mechanism upon which SRGN operates to induce UPR activation, and whether UPR function is contingent upon intracellular, extracellular SRGN or both. A probable model for its mechanism of action could identify SRGN as an extracellular ligand tethering upon a transmembrane receptor to initiate cell signaling and activate UPR signaling as an upstream regulator, together with parallel signal transducers. Nevertheless, our results highlight SRGN as a novel regulator of the UPR pathway in GBM cells, promoting cellular adaptation and survival under prolonged ER stress.

## Data Availability

The original contributions presented in this study are included in the article/Supplementary Material. Further inquiries can be directed to the corresponding author.

## Supplementary Materials

The following supporting information can be downloaded at: https://www.mdpi.com/article/10.3390/cells15080660/s1, Table S1: Primer sequence used for Real-Time qPCR analysis.
