# Supplementary material for: Serglycin Cooperates with the Unfolded Protein Response Pathway and Inflammation to Drive Glioblastoma Cell Survival
Source: Cells. 2026 Apr 9;15(8):660. doi: 10.3390/cells15080660 (PMC13115332; doi:10.3390/cells15080660)
Supplement: Supplementary file 1 [file cells-15-00660-s001.zip › cells-4194267-supplementary.pdf]

Supplementary Table S1

Table S1. Primer sequence used for Real-Time qPCR analysis.

Gene	Primer sequence (5'-3')	$T_{annealing}$ (°C)
ATF4	F: GCTGTGACTTGTGTGTGTCG<br>R: AGAATGACTCCCATGGCAGC	63
ATF6	F: CAGGAACTCAGGGAGTGAGC<br>R: AACAGCAGGTGATCCCTTCG	62
BAX	F: CTGAGCGAGTGTCTCAAGCG<br>R: CCCCAGTTGAAGTTGCCGTC	60
Bcl-2	F: AAGAGCAGACGGATGGAAAAAGG<br>R: GGGCAAATGCAAGTGAATG	60
Beclin-1	F: CAAGATCCTGGACCGTGTCA<br>R: TGGCACTTTCTGTGGACATCA	60
BiP	F: GTCAGGCGATTCTGGTCATT<br>R: GGTGAAAGACCCCTGACAAA	60
CHOP	F: CAGAGTGGTCATTCCCCAGC<br>R: GCTCGATTTCCTGCTTGAGC	63
CTSB	F: GGAGGGAGCTTTCTCTGTGT<br>R: CAGTAGGGTGTGCCATTCTC	60
CXCL-1	F: CTGAGGAGCCTGCAACATGC<br>R: TGATCTCATTGGCCATTTGCTT	60
eIF2a	F: AAAGCTGCAAAGCAGGAAGC<br>R: AGCTGTTTTCCAGTTGCTGC	60
IL-1β	F: GGGCAAGAAGTAGCAGTGTCTGTAAA<br>R: AGAGAGCACACCAGTCCAAATTGA	60
IL-8	F: CTCCAAACCTTTCCACCCC<br>R: GATTCTTGGATACCACAGAGAATG	58
IRE1	F: ACACATGTGGAAGAGCCTGC<br>R: ATCTGAACTTCGGCATGGGG	62
Nrf2	F: AGCCCAGCACATCCAGTCAG<br>R: TGCATGCAGTCATCAAAGTACAAA	60
PERK	F: ACTGTGGAGGACGCTGAGG<br>R: AATTACTAATGACCTGCCGCG	62
TLR2	F: CTCGGAGTTCTCCCAGTTTCTC<br>R: GTCCAGTGCTTCAACCCACA	60
TLR4	F: TGGAAGTTGAACGAATGGAATGTG<br>R: ACCAGAACTGCTACAACAGATACT	60
TNFRI	F: TATTGGACTGGTCCCTCACC<br>R: GTCATTGTACAAGTAGGTTC	60
TNFRII	F: GAACCAGCCACAGGCACCA<br>R: ACGATGCAGGTGACATTGAC	60
s-XBP1	F: GCTGAGTCCGCAGCAGGT<br>R: CTGGGTCCAAGTTGTCCAGAAT	62
u-XBP1	F: CAGACTACGTGCACCTCTGC<br>R: CTGGGTCCAAGTTGTCCAGAAT	62
t-XBP1	F: TGAAAAACAGAGTAGCAGCTCAGA<br>R: CCCAAGCGCTGTCTTAACTC	62
18S rRNA	F: CAGGTCTGTGATGCCCTTAGA	60

	R: GCTTATGACCCGCACTTACTG	
